# Electrospun Nanofibers: from Food to Energy by Engineered Electrodes in Microbial Fuel Cells

**DOI:** 10.3390/nano10030523

**Published:** 2020-03-14

**Authors:** Giulia Massaglia, Francesca Frascella, Alessandro Chiadò, Adriano Sacco, Simone Luigi Marasso, Matteo Cocuzza, Candido F. Pirri, Marzia Quaglio

**Affiliations:** 1 Department of Applied Science and Technology (DISAT), Politecnico di Torino, Corso Duca degli Abruzzi 24, 10129 Torino, Italy; francesca.frascella@polito.it (F.F.); alessandro.chiado@polito.it (A.C.); simone.marasso@polito.it (S.L.M.); matteo.cocuzza@infm.polito.it (M.C.); fabrizio.pirri@polito.it (C.F.P.); 2 Center for Sustainable Future Technologies (CSFT)@Polito, Istituto Italiano di Tecnologia, Environment Park, Building B2 Via Livorno 60, 10144 Torino, Italy; adriano.sacco@iit.it; 3 IMEM-CNR, Parco Area delle Scienze 37, 43124 Parma, Italy

**Keywords:** electrospun nanofibers, polyethylene oxide nanofibers PEO-NFs, microbial fuel cells, honey, food industry, recovered energy (Erec)

## Abstract

Microbial fuel cells (MFCs) are bio-electrochemical devices able to directly transduce chemical energy, entrapped in an organic mass named fuel, into electrical energy through the metabolic activity of specific bacteria. During the last years, the employment of bio-electrochemical devices to study the wastewater derived from the food industry has attracted great interest from the scientific community. In the present work, we demonstrate the capability of exoelectrogenic bacteria used in MFCs to catalyze the oxidation reaction of honey, employed as a fuel. With the main aim to increase the proliferation of microorganisms onto the anode, engineered electrodes are proposed. Polymeric nanofibers, based on polyethylene oxide (PEO-NFs), were directly electrospun onto carbon-based material (carbon paper, CP) to obtain an optimized composite anode. The crucial role played by the CP/PEO-NFs anodes was confirmed by the increased proliferation of microorganisms compared to that reached on bare CP anodes, used as a reference material. A parameter named recovered energy (Erec) was introduced to determine the capability of bacteria to oxidize honey and was compared with the Erec obtained when sodium acetate was used as a fuel. CP/PEO-NFs anodes allowed achieving an Erec three times higher than the one reached with a bare carbon-based anode.

## 1. Introduction

Microbial fuel cells (MFCs) are bio-electrochemical devices that directly convert the chemical energy embedded in an organic compound (i.e., the fuel) into electrical energy by the metabolic action of a particular class of microorganisms, named exo-electrogens.

Basically, the process is based on the ability of exo-electrogens to oxidize organic matter, acting as carbon energy sources [1], and to directly transfer the produced electrons outside their cells exogenously [2]. In an MFC, electrons are firstly released to the anode by the microorganisms arranged in a biofilm in intimate contact with the anode surface and successively they flow through an external circuit to reach the cathode side, where the terminal electron acceptor (TEA), usually oxygen, is finally reduced. Potential applications of MFCs have been foreseen in several areas [3,4], ranging from energy harvesting [5,6] to wastewater treatment [7] and sensing [8,9,10,11,12]. Since MFCs can operate their energy recovery and conversion processes starting from a wide range of molecules, even quite complex, during the last decades, they have attracted an ever-increasing interest for application in the food industry [13]. In this perspective, MFCs can contribute to the overall energetic efficiency of the process by combining the treatment of wastewater streams to energy production. Interesting examples exist, concerning applications in the treatment of brewery wastewater [14,15], olive mill wastewater [16], winery wastes [17,18], and dairy wastewater [19].

The use of MFC for wastewater treatment plays a crucial role as an alternative to traditional treatment processes, such as anaerobic digestion with methane fermentation, which include indirect energy recovery from wastes [20]. Knowledge in this area has grown significantly, and information about the amount of energy than can be recovered from many substrates, such as urban wastewaters [21], short-chain volatile fatty acid [22], as well as fermentable and non-fermentable reference substrates, such as sodium acetate and glucose, respectively, is now available [23].

Nonetheless, further improvements are necessary to increase power production [24], which is currently hindering the marketability of MFCs. In this view, the anodic electrode and its interface with the bacteria biofilm play a crucial role, since they are responsible for energy conversion and the electron transfer process.

In the present work, a new nanofiber-based interface between a carbon paper (CP) anode and a bacterial biofilm is investigated with the aim to optimize the adhesion of the biofilm to the anode. Furthermore, optimized adhesion of the biofilm to the anode plays a crucial role in improving the biofilm–anode electrochemical interaction, thus effectively ameliorating the overall MFC performance in terms of energy production. The idea is based on the hypothesis that the larger is the number of bacteria on the anode, the higher is the resulting electron transfer rate, which is true only if the interface is designed in such a way to keep electrical resistance low. Therefore, in the present work, new nanostructured polymeric mats with high specific area were designed to modify the surface of carbon-based materials in order to improve their ability to create effective interfaces with bacterial biofilms.

During the last decades, some works in the literature [25,26,27] investigated the modification of carbon-based electrodes by the application of a polymeric matrix on their surfaces to increase the contact between bacteria and anodes in MFCs. Several polymeric matrices have been developed, using both natural polymers, such as agar, alginate, or agarose, and synthetic polymers [28,29,30]. Despite being interesting because of their sustainable origin and biocompatibility, natural polymers suffer from poor mechanical strength and durability, while synthetic polymers show an opposite behavior, since they combine higher mechanical strength and durability with a higher risk of toxicity for bacteria proliferation. Among the synthetic polymers proposed up to now in the literature, a promising one is poly(vinyl) alcohol (PVA) [25,26,27]. It has been investigated in different arrangements, as foams, nanofibers, and film, with the aim to reach a high density of immobilized microorganisms while ensuring good mass transfer properties. Currently, the best performing PVA anode-to-biofilm interface system is the one proposed by Bai et al. [27]. They developed a highly porous foam that showed good results in terms of immobilization of microorganisms, but the fabrication process required the use of boric acid, which caused side problems, such as agglomeration of PVA beads and residuals of toxic boric acid and enhanced swelling of the polymer foam. In order to overcome the limits of such technological approach, in the present work, polyethylene oxide nanofibers mats (PEO-NFs) are proposed as a biomass carrier. Electrospinning is selected as the process to obtain the highly porous matrix, and PEO works as the reference polymer. PEO shows a wide range of intriguing properties: (1) it is not cytotoxic, therefore it allows bacteria proliferation, (2) it is a sustainable and environmentally friendly material that can be processed using water as the only solvent, and (3) it is an important solid polymer electrolyte in electrochemical applications [28,29].

PEO-NFs were directly deposited onto CP-based electrodes, according to the electrospinning-on- electrode process, a binder-free method for nanofiber assembly onto an electrode that we introduced in our previous work [30].

The resulting anodes, named CP/PEO-NFs, were investigated in single-chamber MFCs (SCMFCs) and compared with reference devices using pure CP as anodes [30].

We demonstrate a huge improvement of microorganisms’ proliferation toward the desired optical density (OD), thus confirming the possibility to use CP/PEO-NFs as a biomass carrier for bacteria entrapment. CP decorated by PEO-NFs interfaces were then tested in SCMFCs. A mixed bacterial consortium extracted from a marine sediment sample was used as the inoculum source [31]. A current density as high as 12 mA m^−2^ was reached when CP/PEO-NFs was used as the anode electrode. This value is higher than the one reached with CP anodes, indicating how the presence of PEO-NFs interfaces does not affect the electron transfer process, simultaneously inducing the proliferation of microorganisms.

Finally, we tested the potential of the newly designed anodic electrode to be used in MFCs for energy recovery in the agro-food industry. To demonstrate the robustness of our nanofiber-based interface, we selected honey as a new and more complex electron donor than those tested up to now in MFCs. The use of honey by humans traces back to ancient times, and today honey is a crucial ingredient in several products ranging from foods to beverages, as well as in medical products and cosmetics [32,33]. Honey can be classified as a natural sweetener with a complex composition [34]. In particular, honey is a saturated-sugar solution, where the carbohydrates amount to 95% of its chemical components. Moreover, other important compounds are naturally contained into honey, such as proteins, amino acids, enzymes, organic acids, minerals, and vitamins [34,35,36].

Given both its complex composition, that makes it a complete food from a nutritional point of view, and its natural antibiotic behavior, it is quite interesting to analyze the behavior of MFCs fed with water streams containing a limited amount of honey. Indeed, since MFCs’ power production is based on the metabolic activity of exo-electrogenic microorganisms, all substances able to alter and/or modify the biofilm metabolism could alter the power output. Therefore, we tested the newly designed CP/PEO-NFs anodes in SCMFCs in which a honey-in-water solution at 0.02 wt % was used as the electrolyte. Given the aforementioned antibiotic behavior of honey, the first goal of this study was to demonstrate that MFCs can convert the organic matter of honey into electricity without damaging the biofilms, and the second goal was to demonstrate that new nanofiber-based anodes are more efficient than the commonly used CP references.

Results are analyzed in terms of recovered energy (E_rec_) per unit volume [18,21,37]. E_rec_ was obtained by the integration of the measured power output over the batch treatment time. In particular, E_rec_ obtained for honey was compared with E_rec_ obtained when sodium acetate was used as a fuel in SCMFCs, demonstrating the microorganisms’ capability to oxidize honey. A further comparison is then proposed to appreciate the different behavior of CP/PEO-NFs anodes in comparison to CP reference anodes.

Finally, we demonstrate that the SCMFCs’ response, in terms of power output, changes with honey concentration. Different concentrations of honey (from 0.83 to 2 gL^−1^) were tested and correlated with the corresponding energy recovery parameter.

## 2. Materials and Methods

### 2.1. Materials and Nanofibers Synthesis

A new nanofiber-based interface between a carbon paper-based anode and a bacteria biofilm was investigated. In particular, new nanostructured polymeric mats with high specific area were designed to modify the surface of carbon-based materials. Nanofibers mats based on PEO, were directly electrospun on a carbon-based material (named CP, Fuel Cell Earth, Woburn, Massachusetts, USA), used as reference material since it is the most employed anode in MFCs. As already demonstrated in our previous work [30], the morphology of CP, characterized by several conductive micro-protrusions, plays a crucial role in tuning a selective patterned deposition of PEO nanofibers (CP/PEO-NFs), thus leading to ensure a binder-free deposition of nanofibers. In particular, CP/PEO-NFs were obtained by electrospinning, starting from a polymeric solution containing PEO (purchased from Sigma Aldrich, with an average molecular weight Mw = 600 kDa) dissolved in deionized water. Electrospinning was performed by the NANON 01A machine from MECC Co. Ltd. The polymeric solution was loaded into a syringe, and nanofiber were obtained by applying a high positive voltage equal to 20 kV and a flow rate of 0.5 mL h^−1^ at a working distance of 12 cm. The duration of the electrospinning process was close to 10 min to ensure at the same time an ordered distribution of nanofibers, which were directly collected onto CP without a binder, and a high surface area-to-volume ratio. As demonstrated in our previous work [30], the ordered distribution is mainly due to the conductive protrusions of CP that induce electric field enhancement. This ordered distribution can be optimized when the thickness of the nanofibers is close to few micrometers. Indeed, a higher thickness of nanofiber mats could induce an insulator effect onto a carbon-based surface, minimizing the electric field variations that rule the nanofibers’ distribution onto a CP surface. Moreover, in the present work, the surface area was defined by implementing Brunauer–Emmett–Tell (BET) measurements. In order to establish the lack of cytotoxicity of PEO for microorganisms, optical density (OD) measurements were carried out by means of a LAMBDA 850+ UV/Vis spectrophotometer. 

In particular, OD measurement estimated the growth and metabolic activity of bacteria. Both CP/PEO-NFs and bare CP pieces were put into a tube containing the inoculum source and the electrolyte solution. The electrolyte solution was based on sodium acetate (C_2_H_3_NaO_2_), ammonium chloride (0.31 gL^−1^ of NH4Cl) used as a nitrogen source to aid bacteria growth, and phosphate-buffered solution (PBS) that maintained a neutral pH. Every day, total cell density (dead and alive cells) was established by measuring OD at 600 nm using the spectrophotometer.

### 2.2. MFC Architecture and Configuration

The MFCs used in the present work were squared single-chamber microbial fuel cells (SCMFCs) with an open-air cathode, fabricated by micro milling (Al.Tip srl). [38]. In particular, our devices were composed of 3 compartments: the anodic part, the intermediate compartment, and the cathodic compartment. The internal volume of SCMFCs was 12.5 mL, and both anode and cathode had a geometric area equal to 5.76 cm^2^. Furthermore, in the present work, two different anodes were investigated and compared: (1) CP/PEO-NFs obtained by direct deposition of PEO nanofiber mats on a carbon-based electrode, employed as a carbon backbone to ensure the electron transfer generated and released by the microorganisms; (2) a carbon-based material (CP) used as a control. The cathode was based on CP, properly modified in order to present gas diffusion layers (DLs) based on polytetrafluoroethylene (PTFE) on its outer side and a catalyst layer based on Platinum (Pt/C 0.5 mg/cm^2^, from Sigma Aldrich, St.Louis, Missouri, USA) and Nafion (5 wt % Nafion, from Sigma Aldrich) on its inner side. Titanium wires (Goodfellow Cambridge Limited) were fixed onto the anode and cathode through a conductive paste made of carbon cement (Leit-C Cement). Two different electrolyte solutions were used. The first was an electrolyte solution based on sodium acetate (C_2_H_3_NaO_2_), used as a carbon energy source at a concentration of 2 gL^−1^ together with other compounds able to ensure the optimal operation of the SCMFCs. All these compounds were based on ammonium chloride (0.31 gL^−1^ of NH4Cl), used as a nitrogen source to support bacterial growth, and PBS, able to maintain a neutral pH (containing 0.13 gL^−1^ of potassium chloride, 4.28 gL^−1^ of sodium phosphate dibasic, and 2.45 gL^−1^ of sodium phosphate monobasic monohydrate). The second electrolyte contained honey in the same amount defined for sodium acetate, close to 2 gL^−1^, dissolved in deionized water and PBS. In the second electrolyte, PBS was added to ensure a neutral pH of the solution; due to the complexity of honey, the second electrolyte did not require any further addition of nutritional compounds for the microorganisms. Both solutions were autoclaved prior to use. All experiments were conducted in duplicate for each tested electrolyte: 2 SCMFCs with CP/PEO-NFs anode and 2 SCMFCs with CP anodes were studied. All SCMFCs were inoculated with a mixed culture of bacteria from a seawater sediment. All SCMFCs worked under batch mode, meaning that all devices are filled with *“new electrolyte”* when the drop of power output reached its minimum value. Anodes and cathodes of the SCMFCs were connected to a multichannel data acquisition unit (Agilent 34972A), and two different values of external resistance were applied. At the beginning of the experiments, to ensure the formation of biofilm on both anodes, i.e., CP/PEO-NFs and CP, an external load equal to 47 Ω was applied, and the electrolyte containing sodium acetate was used. Successively, to evaluate the overall SCMFCs’ performance and to demonstrate that SCMFCs can convert honey organic matter into electricity without damaging the biofilms, an external load of 1000 Ω was applied. Furthermore, to establish and confirm the possibility to employ honey as a fuel in SCMFCs and demonstrate that its natural antibiotic behavior does not affect the overall SCMFCs’ performance, we introduced a physical parameter, i.e., energy recovery (E_rec_), calculated for both honey and sodium acetate [18,21,37]. The E_rec_ was defined according to Equation (1):

(1) Erec=∫ PdtVint

where *E_rec_ (J m^−3^)* is energy recovery, $V_{int}$(m^3^) is the internal volume of SCMFCs, and $\int^{\;}Pdt$ (J) is the integral of the generated power over time. We were able to demonstrate the possibility of using honey as a fuel in SCMFCs. In order to simultaneously verify the SCMFCs’ response in terms of power output as honey concentration changed, different honey concentrations (from 0.83gL^−1^ to 2 gL^−1^) were tested and correlated with the values of power output. 

Internal resistance of the SCMFCs was evaluated through Nyquist plots, using electrochemical impedance spectroscopy (EIS). EIS was performed in open-circuit voltage (OCV) over the range of frequency between 150 and 200 mHz, with a sinusoidal signal with an amplitude of 25 mV.

## 3. Results and Discussion

### 3.1. Electrospun Nanofibers Onto Carbon-Based Materials and Their Role as a Biomass Carrier

**Figure 1:**
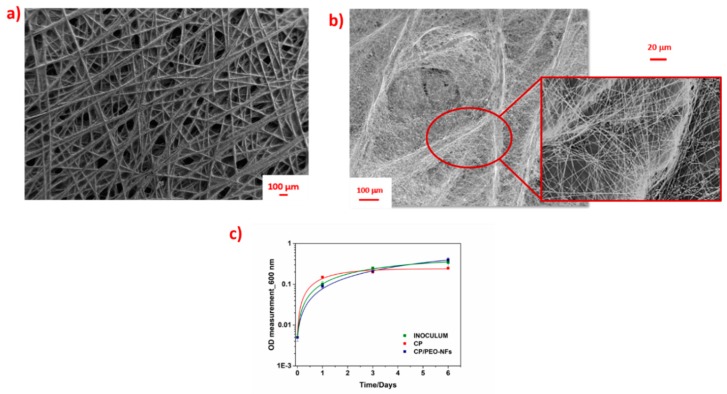
(**a**) FESEM images representing the bare carbon-based material (CP); (**b**) FESEM images representing the ordered distribution of CP/polyethylene oxide nanofibers (PEO-NFs) onto the carbon-based material. In the red box, the preferential distribution of CP/PEO-NFs onto the conductive protrusions of CP is highlighted; (**c**) logarithmic representation of OD measurements performed for all samples: bare inoculum (green), carbon-based material (CP, red), and CP/PEO-NFs (blue).

As already demonstrated in our previous work [30] and in order to optimize the deposition of nanofiber mats onto carbon-based materials, we selected CP as a carbon-based material. The morphology of bare CP is reported in Figure 1a. In particular, it is possible to appreciate that the morphology of CP shows many naturally occurring conductive protrusions, based on carbon fibers, whose diameter was over 10 µm. All these protrusions, as also demonstrated in our previous work [30], play a crucial role in modulating the electric field during the electrospinning process, inducing an intensity enhancement and granting an ordered distribution of PEO-NFs. As represented in the Figure 1b, CP/PEO-NFs nanofibers preferentially arranged themselves on top of the conductive protrusions of CP, thus optimizing the interface between the nanofibers mats and the carbon-based electrode without the presence of a binder, commonly used to fix the nanostructures onto the electrode. 

Figure 1a,b shows that PEO-NFs were mostly deposited onto the CP surface in correspondence of the interconnections between the conductive protrusions, covering completely the CP surface. Moreover, the final CP/PEO-NFs anode resulted to be an engineered electrode with a high surface area-to-volume ratio. As indicated by the BET results, PEO-NFs showed a surface area close to 40 m^2^ g^−1^. 

As presented in Figure 1c, the OD measurements confirmed the capability of CP/PEO-NFs to create an effective interface with bacterial biofilms, thus ensuring bacterial proliferation onto the CP-based anode. This result also confirmed the possibility to apply CP/PEO-NFs as a biomass carrier. The high bacterial proliferation is due to the capability exhibited by CP/PEO-NFs to entrap the microorganisms into its nanostructures.

### 3.2. SCMFCs Performance

**Figure 2:**
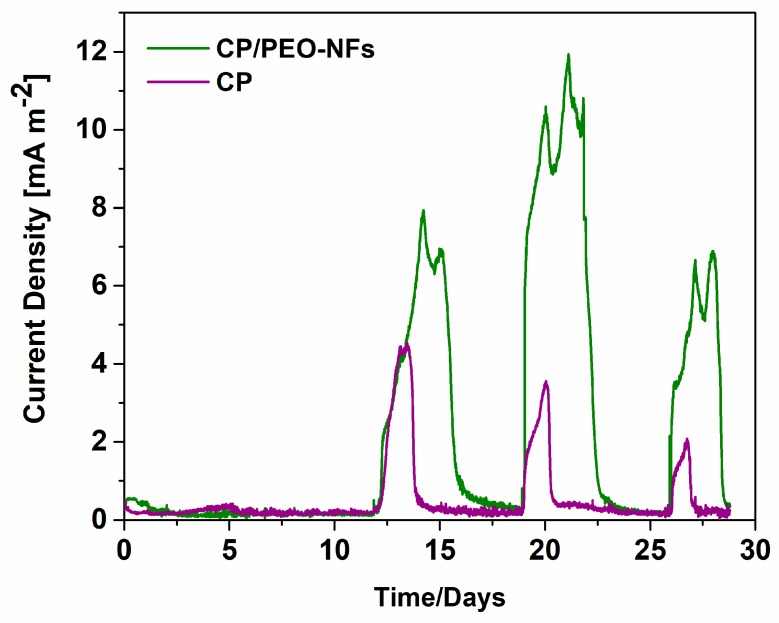
Current density produced in the acclimation phase, during which the biofilm formed onto the anodes: CP/PEO-NFs (green line) and carbon-based material used as a reference electrode (purple line). In this phase, the electrolyte contained sodium acetate; an external load of 47 Ω was applied.

Thanks to CP/PEO-NFs biocompatibility, in terms of microorganisms’ proliferation and their morphological properties, CP/PEO-NFs mats were produced and directly deposited onto the carbon-based material. During the first phase of the experiment, known as the acclimation phase, we applied an external resistance close to 47 Ω with the main aim to ensure biofilm formation onto the electrodes. The duration of this acclimation phase was 1 month. As shown in Figure 2, the overall performance reached by the CP/PEO-NFs composite anode was three times higher than the one obtained with the bare carbon-based material. Since all the other features, i.e., the cathode, SCMFCs’ configuration, and the electrolyte, were the same, it was possible to establish that the enhancement of the current density was related to the proliferation extent of the microorganisms, being higher onto PEO–NFs composite anodes. Moreover, by analyzing all peaks reported in Figure 2, it was possible to demonstrate a good electrical output produced by SCMFCs and, consequently, a stable and sustained bacteria proliferation on the anodes.

**Figure 3:**
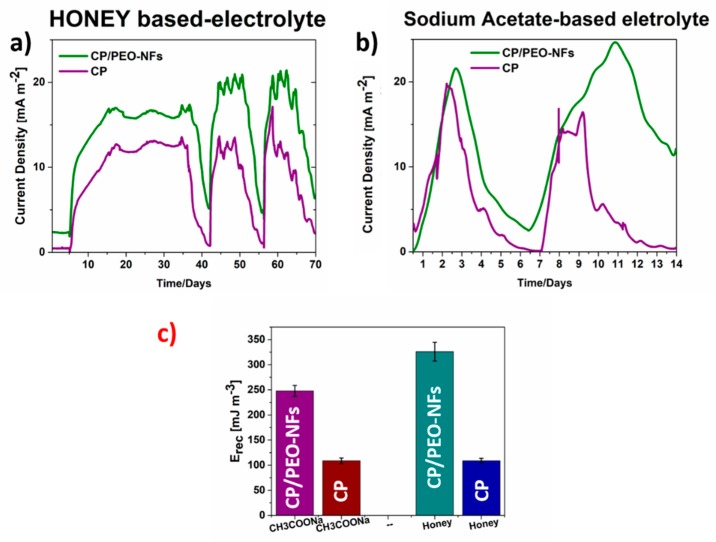
(**a**) Current density obtained when honey was used as a fuel and comparison of the current density values obtained for CP/PEO-NFs and CP; (**b**) current density reached using sodium acetate as a fuel and comparison of the values obtained for CP/PEO-NFs and CP; (**c**) recovered energy (E_rec_) determined for CP/PEO-NFs and CP, using two different fuels.

The overall performance obtained with CP/PEO-NFS was compared with that of a reference material consisting of bare carbon-based anodes (CP). Moreover, given the complex composition of honey and its natural antibiotic behavior, a second experiment was implemented to demonstrate that SCMFCs can convert honey organic matter into electricity without damage to a biofilm and to confirm that the new nanofiber-based anodes (CP/PEO-NFs) perform better than the CP reference anodes. In this experiment, an external load of 1kΩ was applied, and the overall performance, when both electrolytes were used, was analyzed. In this case, the concentration of sodium acetate and honey was 2 gL^−1^. The current density trends, obtained from all tests in SCMFCs, were defined by normalizing with respect to the anode geometric area (5.76 cm^2^) and are reported in Figure 3a,b. It is possible to appreciate that the maximum current densities reached when sodium acetate and honey were used as electrolytes were comparable, demonstrating the capability of bacteria to catalyze the oxidation reaction of honey. These results also suggest the possibility to apply SCMFCs to convert honey organic matter into electricity without damaging the biofilms. Moreover, since all these devices exploited formally identical cathodes and architectures, the current density trends were univocally correlated with the anodic reaction. Furthermore, as presented in Figure 3a,b, the maximum current density of total SCMFCs, reached when CP/PEO-NFs were used as the anode, was equal to (23.2 ± 0.1) mA m^−2^ and was comparable to the one obtained when CP was used as the reference anode. The presence of PEO-NFs, which basically showed insulating properties from an electrical point of view, not only did not affect the electron transfer rate but also simultaneously sustained the proliferation of microorganisms onto the anode. Moreover, all results are analyzed in terms of recovered energy (E_rec,_ mJ m^−3^). E_rec_ is calculated by integrating the electric power density over the batch treatment time and normalizing with respect to the electrolyte volume. Figure 3c shows that E_rec_ (equal to 100 mJ m^−3^) obtained when using honey and CP material was close to E_rec_ reached when sodium acetate was employed as the electrolyte. Moreover, for both fuels, E_rec_ achieved when using CP/PEO-NFs was three times higher than the one reached when using the carbon-based material, confirming the crucial role of nanofibers, which ensure a better interface between the anode and the bacteria and enhance the overall performance of SCMFC. This latter result demonstrates that PEO-NFs can act as a biomass carrier. The interface between biofilm and CP/PEO-NFs anode was enhanced, as also confirmed by the OD measurements, underlying a more extensive bacterial proliferation onto CP/PEO-NFs anodes. Both features, the optimized interface between anode and biofilm and the role of PEO as a polymeric electrolyte in electrochemical devices, allowed reaching a higher E_rec_ in comparison with the one reached when using a bare carbon-based material (CP).

**Figure 4:**
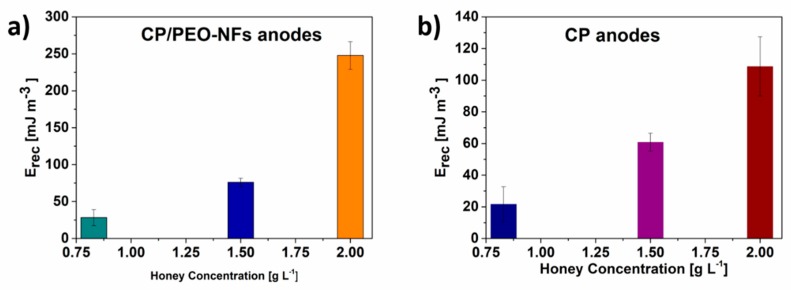
(**a**) Recovered energy defined for CP/PEO-NFs correlated with honey concentration; (**b**) Recovered energy defined for CP correlated with honey concentration.

Figure 4a,b show that the performance of all devices increased with increasing honey concentrations from 0.83 gL^−1^ to 2 gL^−1^. The maximum value of recovered energy E_rec_ was reached at the highest honey concentration employed (2 gL^−1^) for both CP/PEO-NFs and CP anodes. At all honey concentrations, CP/PEO-NFs showed a better performance in terms of E_rec_ than the CP anodes.

### 3.3. Electrochemical Impedance Spectroscopy Results

**Figure 5:**
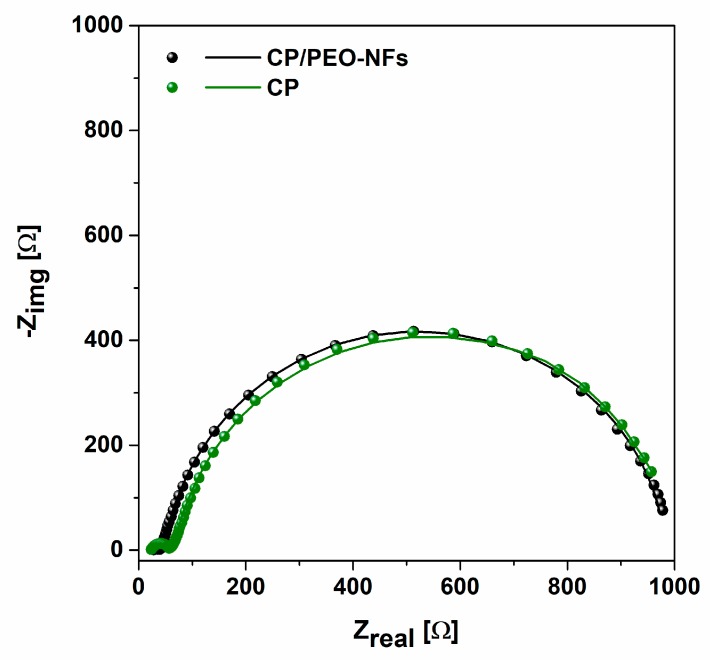
Impedance spectra of CP/PEO NFs (black dots and line, representing the experimental data and their fitting) and CP anode (green dots and line).

EIS was performed to investigate the impedance behavior and, in particular, the internal resistance [39] when honey was employed as a fuel. By analyzing the Nyquist plot represented in Figure 5, it can be observed that MFCs exploiting CP/PEO-NFs as the anode were characterized by similar total impedance values with respect to carbon-based devices (982.2 Ω and 916.5 Ω, respectively). Moreover, the results allowed analyzing the resistance related to the charge transfer (R_ct_) of both CP/PEO-NFs and CP anodes. The obtained results confirmed that SCMFCs exploiting CP/PEO-NFs as the anode presented impedance values close to those reached with a CP-based anode. The value of R_ct_ for CP/PEO-NFs, close to 761 Ω, was quite similar to that obtained for CP, equal to about 808 Ω. These results demonstrated that the presence of PEO-NFs, which basically has insulating properties from an electrical point of view, not only did not affect the electron transfer rate but also simultaneously sustained the proliferation of microorganisms onto the anode 

This result suggests the possibility to apply CP/PEO-NFs to achieve a large nanofiber-based interface between a CP anode and a bacterial biofilm, thus optimizing the adhesion of the biofilm to the anode.

## 4. Conclusions

In the present work, a CP/PEO-NFs composite anode, based on PEO-NFs directly electrospun onto a carbon-based material, was designed and optimized. CP/PEO-NFs showed a final ordered arrangement of PEO-NFs, able to grant a large surface area-to-volume ratio. We demonstrated that the resulting structure of PEO/NFs greatly promoted microorganisms’ proliferation, thus suggesting the possibility to employ PEO-NFs as a biomass carrier for bacteria entrapment. Therefore, we investigated the behavior of a CP/PEO-NFs anode in SCMFCs, using a mixed bacterial consortium extracted from a marine sediment sample as the biofilm source. In particular, a high current density, close to 20 mA m^−2^, was reached when CP/PEO-NFs were used as the anode. This value, higher than the one reached when bare CP was used as the anode, allowed us to demonstrate that the presence of PEO-NFs, with their insulating properties from an electrical point of view, did not affect the electron transfer rate and simultaneously sustained the proliferation of the microorganisms. Finally, the results concerning the evaluation of energy recovery confirmed the possibility to use a more complex substrate, such as honey, as a fuel in MFCs. Moreover, we evidenced that the new designed CP/PEO-NFs anodes are able to ensure a three-time higher recovered energy (i.e., 300 mJ m^−3^) than that obtained when using a bare carbon-based anode.

The results show that nanostructured interfaces made of PEO-based nanofibers are advantageous for the fabrication of robust and efficient electrodes to be used in MFCs as energy conversion tools for the valorization of waste in the food industry.
